# Quorum Quenching and Quorum-Sensing Interference in *Pseudomonas aeruginosa*: Molecular Mechanisms, Host Protection, and Translational Barriers

**DOI:** 10.3390/ijms27219439

**Published:** 2026-10-23

**Authors:** Xin Zhao, Wei Zou, Leilei Pei

**Affiliations:** 1 Department of Epidemiology and Health Statistics, School of Public Health, Xi’an Jiaotong University Health Science Center, No. 76 Yanta West Road, Xi’an 710061, China; xinxinxin928@163.com; 2 Infection Control Office, Xi’an Public Health Center (Xi’an Emergency Medical Center), Xi’an 710200, China; 3 Yunnan Key Laboratory of Cross-Border Infectious Disease Prevention and New Drug Development, Kunming Medical University, Kunming 650500, China; 4 Yunnan Provincial Key Laboratory of Public Health and Biosafety & School of Public Health, Kunming Medical University, Kunming 650500, China

**Keywords:** quorum quenching, quorum sensing, *Pseudomonas aeruginosa*, anti-virulence therapy, biofilm, host protection, antibiotic tolerance, drug delivery

## Abstract

Quorum sensing (QS) coordinates virulence expression, biofilm-associated behavior, and persistence in *Pseudomonas aeruginosa*, making its signaling network a potential target for adjunctive anti-virulence therapy. Quorum quenching (QQ) directly degrades, modifies, or sequesters signaling molecules, whereas broader QS interference encompasses inhibition of signal synthesis, receptor antagonism, and disruption of downstream regulatory interactions. Experimental studies of N-acyl-homoserine lactone (AHL)-degrading enzymes and PqsR antagonists have demonstrated suppression of selected virulence-associated phenotypes, with several infection models showing reductions in host injury or persistence-associated outcomes. The PqsE–RhlR regulatory interface has gained substantial molecular and genetic support as a distinct intervention target, but its pharmacological development remains limited. Responses to QS interference vary with bacterial strain, regulatory network adaptation, environmental conditions, and infection stage. Reductions in virulence-factor production or biofilm biomass do not consistently correspond to bacterial clearance or host protection. Available human studies involving agents with proposed QS-modulatory activity have not established clinical benefits specifically attributable to QS inhibition. Clinical translation requires validated target engagement, efficacy assessment in established and polymicrobial infections, and characterization of delivery, pharmacological exposure, safety, and resistance evolution.

## 1. Introduction

### 1.1. Medical Importance of Pseudomonas aeruginosa Infection

*Pseudomonas aeruginosa* is an opportunistic pathogen associated with acute and chronic airway infections, including ventilator-associated pneumonia and infections in patients with cystic fibrosis or bronchiectasis; burn and chronic-wound infections; urinary tract infections; and device-associated infections. Clinically relevant devices include urinary and vascular catheters and respiratory devices, but findings from one device type should not be assumed to apply to others. Reports of catheter coatings and intravesical delivery describe distinct local experimental applications: antimicrobial or anti-adhesive modification of catheter materials and administration into the urinary bladder, respectively [1,2,3,4]. The clinical burden is shaped by difficult source control, intrinsic and acquired resistance, repeated antibiotic exposure, and the ability to persist in biofilms. These settings warrant careful separation of clinical epidemiology from laboratory proof of QS-target engagement [5,6].

During chronic airway infection, prolonged colonization can remodel QS systems. In cystic fibrosis isolate collections, Las-associated signaling can be lost while Rhl-associated outputs persist, and mucoid isolates may retain N-butanoyl-L-homoserine lactone (C4-HSL) production and rhamnolipid-associated phenotypes [7]. The *lasR*-deficient cystic fibrosis isolate E90 retained density-dependent RhlR activity and RhlR-mediated epithelial cytotoxicity; this single-isolate finding requires confirmation in larger clinical collections [5]. Correlative bronchiectasis data and a prophylactic palmitoleic acid mouse model provide additional host context, but neither constitutes therapeutic evidence in humans [8]. Persistence and recurrence are distinct from the immediate bactericidal response. Preclinical studies of PqsR inhibition and phenazine-producing biofilms indicate that persistence, tolerance, recurrence and viable bacterial burden should not be conflated [9]. In preclinical infection models, inhibition of PqsR (also known as MvfR) can reduce persister-associated phenotypes and lower the risk of recurrence [10].

### 1.2. Limitations of Conventional Antibiotic-Centered Treatment

Conventional management depends on infection-site assessment, source control where feasible, susceptibility-guided antimicrobial therapy, and adequate drug exposure. Treatment is complicated by intrinsic permeability barriers, efflux, acquired resistance mechanisms, and resistant subpopulations; antibiotic choices and combinations depend on the infection site and susceptibility pattern rather than QS status alone [11,12,13]. M64 retained anti-virulence activity in multiple multi-drug-resistant isolates [10], whereas SsoPox-W263I reduced selected virulence traits in clinical isolates [1] and phage-resistant strains [2]. These experimental findings suggest possible adjunctive strategies but do not establish clinical efficacy or substitute for effective antibacterial treatment.

Biofilms add physical and physiological barriers. Phenazine-producing PA14 biofilms exhibit markedly increased tolerance to ciprofloxacin, tobramycin and carbenicillin but increased susceptibility to colistin, with no change in planktonic minimum inhibitory concentration (MIC) [9]. Mucin adsorbed a hydrophobic QS inhibitor, while nanoparticle charge and interactions with the biofilm matrix altered penetration and activity in biofilm-relevant systems [14,15]. Antibiotic susceptibility, biofilm tolerance and drug-delivery efficiency are related yet distinct.

Resistance is a heritable reduction in antimicrobial susceptibility, typically reflected by an elevated planktonic MIC, whereas tolerance is survival during antimicrobial exposure without a necessary change in MIC [16]. Phenazine-producing biofilms exemplify tolerance without changes in MIC. In QS mutants, changes in biofilm biomass did not consistently correspond to increased antibiotic susceptibility. Biomass, viable bacterial burden, tolerance and resistance should be treated as independent endpoints [17,18].

Some barriers are independent of QS biology. The MexAB-OprM efflux pump reduced the potency of several QS inhibitors in native *P. aeruginosa*, showing that reporter activity can overestimate efficacy when efflux or insufficient intracellular exposure limits target engagement [12,13]. Improved antibiotic penetration through a formulation does not demonstrate independent bactericidal activity of the QS inhibitor. Treatment failure is multifactorial and involves resistance, tolerance, efflux, matrix composition, metabolic state, host environment and timing of administration.

### 1.3. Rationale for Anti-Virulence Strategies

Anti-virulence therapy targets pathogenic damage rather than direct killing alone [19]. SsoPox-I increased survival and reduced lung pathology in acute rat pneumonia with little change in lung colony-forming units (CFUs); PvdQ reduced pulmonary inflammation and histological injury with model-dependent effects on bacterial burden; and M64 was associated with better host outcomes and reduced persistence or relapse phenotypes without acting as a conventional bactericidal agent [10,20,21]. These results separate host protection from bacterial clearance [22].

Potential intervention outcomes include reduced virulence-factor production, biofilm development, persistence, tolerance, epithelial injury, and inflammation. Signal-degrading enzymes reduced selected virulence outputs and delayed nematode killing, whereas PqsR-directed interventions altered pathway-specific virulence and persistence-associated phenotypes [23,24,25,26]. Genetic and biochemical studies of PqsE–RhlR identified a downstream regulatory interface that affects virulence, but they have not yet defined a mature drug class [26,27,28,29].

Anti-virulence interventions are best viewed as adjuncts, not antibiotic replacements [30]. Studies of QS-directed enzymes or receptor antagonists, alone or in combination with antibiotics or phages, have produced context-dependent effects: PqsR antagonism potentiated ciprofloxacin during one early treatment interval but not in a mature-biofilm assay, and QS disruption did not consistently sensitize a 72 h biofilm under controlled experimental conditions [17,25,26]. Their therapeutic value depends on whether QS interference increases host protection or antibiotic access, promotes clearance, or reduces relapses in a defined setting. Anti-virulence therapy is not inherently resistance-proof. Social-evolution experiments used engineered sensitive and resistant mimics with an idealized inhibitor, rather than a clinical agent under long-term treatment [31,32]. Efflux-mediated potency loss and naturally rewired clinical isolates reinforce this concern.

### 1.4. Quorum Sensing as a Therapeutic Target

The principal Las, Rhl, and Pqs circuits contain experimentally tractable molecular targets, including signal-biosynthetic enzymes, signal-responsive receptors, and downstream protein interactions that could potentially be perturbed [33]. A validated biological target is not necessarily a druggable or clinically validated therapeutic target. LasR, RhlR, PqsR, and the PqsE–RhlR interface regulate overlapping but non-equivalent outputs; active pathways can differ across clinical isolates and host environments [5,7,27,28,34]. Agent selection requires evidence of the relevant active pathway, direct target engagement at achievable exposure levels, and benefit in the intended infection setting [35].

The PqsE–RhlR axis and Pseudomonas quinolone signal (PQS)-associated effects illustrate why a single receptor readout cannot represent the full network. RhlR output is jointly shaped by canonical C4-HSL signaling and PqsE-dependent regulation, with promoter-specific dependencies across the regulon [36,37,38,39]. PQS can exert effects not captured by PqsR transcriptional inhibition, including iron-linked activities [40], whereas PQS-associated outer membrane vesicle biology provides a QS-adjacent route to dispersion and matrix remodeling [39].

### 1.5. Definition and Scope of Quorum Quenching in This Review

In this review, canonical quorum quenching (QQ) denotes direct enzymatic degradation, chemical transformation, or physical sequestration of QS signaling molecules and thereby reduces their availability or signaling activity [41]. Here, “interception” refers to physical signal capture before receptor binding and is included within sequestration rather than treated as a separate biochemical mechanism. Broader QS interference also includes inhibition of signal production, receptor antagonism, and perturbation of defined QS-associated regulatory interactions. Gac/Rsm and c-di-GMP networks are global regulatory systems that can crosstalk with QS but are not, by their nature, direct QQ targets [27,29,42,43]. Reduced QS-gene expression, virulence-factor production, or biofilm staining alone cannot establish direct QQ without mechanistic and matched growth controls [44,45].

### 1.6. Medical and Host-Protective Perspective

Here, “host protection” means a reproducible improvement in a measured host outcome—such as epithelial barrier integrity, tissue pathology, inflammatory injury, morbidity, or survival—relative to an appropriate infected control. It does not imply bacterial eradication, reduced viable burden, or clinical cure. QS-targeted studies should report bacterial biomass, viable CFU, antibiotic tolerance, and host outcomes as separate endpoints, since they may diverge [46].

Delivery and safety add further translational constraints. Mucus adsorption, biofilm penetration, efflux, particle charge, protein corona formation, local deposition and formulation-dependent biofilm effects can determine whether an agent reaches its target [12,14,15,47]. No intervention reviewed here has been characterized across pharmacokinetics, biodistribution, repeated dosing, microbiome effects, immunogenicity, and resistance or pathway escape; recent reviews identify the same gaps [48].

Recent reviews survey QS and QQ strategies across the antibiofilm and anti-virulence literature [15,28,48]. This review takes a narrower, mechanism-centered focus on *P. aeruginosa*. It separates direct signal quenching from signal-synthesis inhibition, receptor antagonism, downstream regulatory interference and QS-adjacent effects, informed by contemporary mechanistic syntheses [48] and the foundational experimental QS literature [49].

### 1.7. Literature Search and Evidence-Selection Approach

This article was prepared as a critical narrative review focusing on the molecular mechanisms, experimental evidence, infection-related outcomes, and translational limitations of quorum quenching (QQ) and broader quorum-sensing (QS) interference in *Pseudomonas aeruginosa*. The relevant literature was identified through targeted searches of PubMed, Scopus, and Web of Science Core Collection, with the final search conducted on 11 October 2026. ClinicalTrials.gov was separately consulted to identify registered human investigations of QS-modulating interventions.

Search terms combined “*Pseudomonas aeruginosa*” with QS-related terms, including “quorum sensing”, “quorum quenching”, “quorum sensing inhibition”, “anti-virulence”, “biofilm”, and “infection”, supplemented by mechanism-specific terms such as “lactonase”, “acylase”, “LasR”, “RhlR”, “PqsR”, “MvfR”, “PqsE”, “PQS”, and “HHQ”. The literature published from 1993 to 11 October 2026 was considered eligible for inclusion to capture the historical development, mechanistic evolution, and current translational status of QS-targeted strategies in *P. aeruginosa*. Studies were included when they provided mechanistic, experimental, or translational evidence related to QS signaling, signal degradation or sequestration, signal-synthesis inhibition, receptor antagonism, downstream regulatory interactions, biofilm-associated phenotypes, host outcomes, or delivery strategies. Original studies reporting direct molecular or biochemical validation, appropriate growth controls, quantitative outcomes, or infection-model evidence were prioritized. Studies lacking direct relevance to QS mechanisms, focusing only on nonspecific antimicrobial activity, or without sufficient information for evidence assessment were excluded. QS-associated phenotypic changes without direct target validation were considered indirect evidence of pathway modulation. Clinical investigations were evaluated separately to distinguish therapeutic outcomes from mechanisms proposed based on preclinical studies. Evidence was assessed according to four dimensions: mechanistic validation, experimental quality, infection-model relevance, and translational readiness. The literature was synthesized qualitatively without formal systematic-review screening or quantitative meta-analysis. Because narrative synthesis may introduce selection bias, predefined evidence-evaluation criteria were applied to improve transparency. Statements regarding AHL-degrading enzymes and selected PqsR-targeted interventions therefore reflect their relatively stronger combination of mechanistic validation and preclinical host-level evidence among evaluated studies, rather than superiority over other QS-targeted approaches. Key statements were cross-checked against multiple independent studies where available, and conclusions were weighted according to the consistency of mechanistic evidence and infection-model validation rather than publication counts.

## 2. The Quorum-Sensing Network of *Pseudomonas aeruginosa* and Its Pathogenic Significance

### 2.1. Core Components of the Las, Rhl, and Pqs Systems

**Figure 1:**
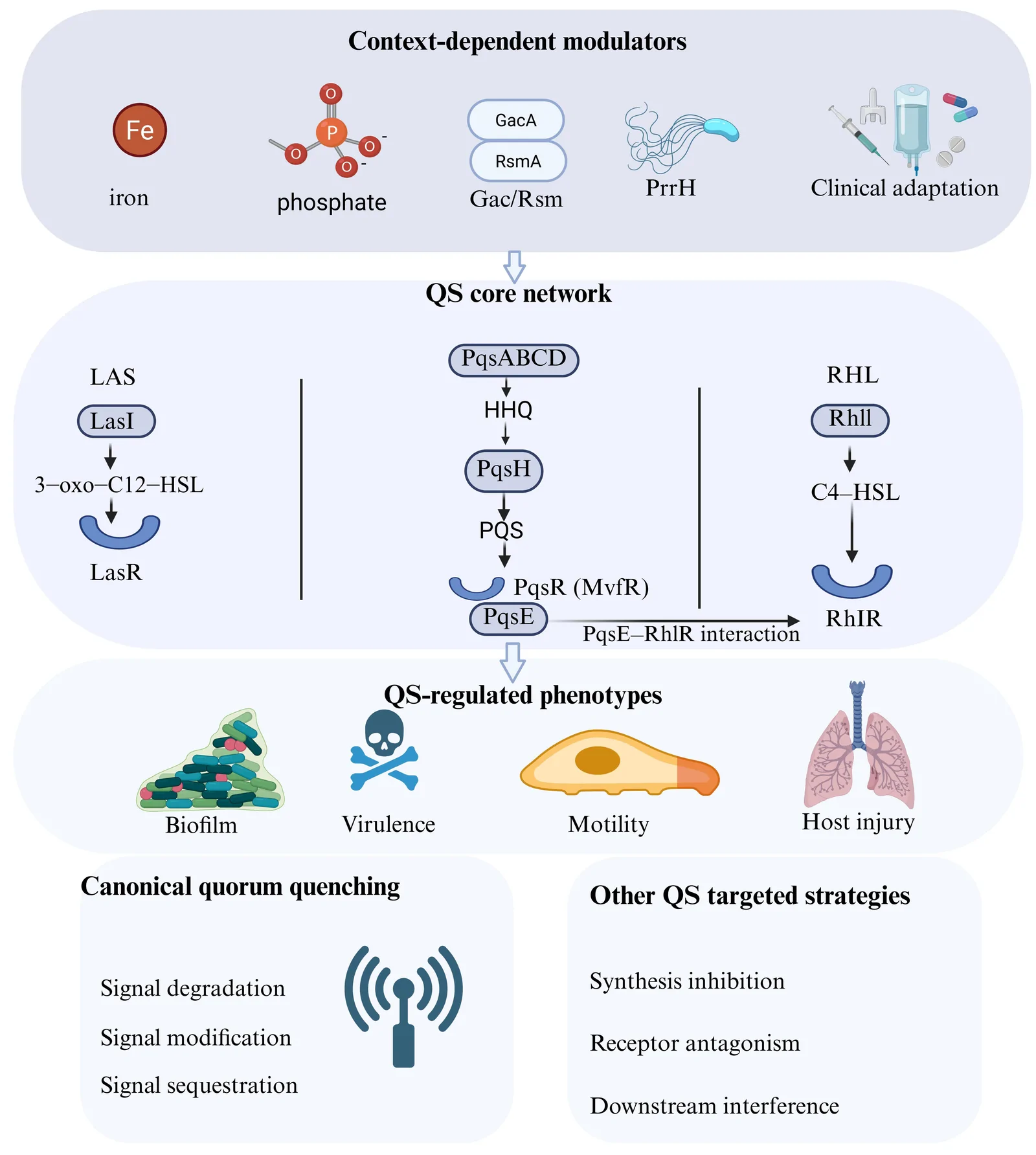
Quorum-sensing (QS) network and candidate intervention points in *Pseudomonas aeruginosa*. LasI produces 3-oxo-C12-HSL for LasR, while RhlI produces C4-HSL for RhlR. PqsABCD supports HHQ biosynthesis and PqsH converts HHQ to PQS; PqsR (MvfR) regulates alkylquinolone-related transcription. The PqsE–RhlR link denotes a functional protein–protein interaction, not enzymatic conversion of PQS into PqsE. Arrows denote the labeled biosynthetic or functional relationships; iron, phosphate, Gac/Rsm, PrrH and clinical adaptation are context-dependent modifiers. PqsR activation drives pqsABCDE transcription, whereas PqsE acts through a distinct regulatory interaction with RhlR; the adjacent placement of these proteins does not denote enzymatic conversion or direct processing.

LasI and RhlI synthesize 3-oxo-C12-HSL and C4-HSL, respectively; these signals activate their corresponding receptors, LasR and RhlR [33]. The Pqs circuit has distinct biosynthetic, receptor-mediated, and downstream regulatory layers [50,51,52]. The PqsABCD-dependent pathway contributes to HHQ biosynthesis; PqsH mediates conversion of HHQ into PQS. PqsR (MvfR) regulates alkylquinolone biosynthetic genes, while PqsE can shape virulence through its functional interaction with RhlR [27,53,54]. PQS also has receptor-independent activities, including iron-related and outer-membrane-vesicle-associated effects [39,40,55]. Figure 1 presents these regulatory layers separately and does not imply a linear PqsH → PqsABCD → PqsR → PqsE cascade. Global Gac/Rsm regulation is related to, but distinct from, the direct signaling steps [43,56]. Canonical QQ acts directly on signal availability; other QS-directed interventions act at synthesis, receptor or downstream regulatory levels [27,53,54,57].

The Pqs system is not a linear switch [50,51]. PqsR principally activates the *pqs* biosynthetic program, PqsE controls broader virulence and biofilm-associated outputs, and PQS has receptor-independent effects linked to iron homeostasis and secretion-associated functions [52]. Suppressing PqsR-associated transcription does not necessarily block PqsE–RhlR biology or PQS-associated physical effects. Las, Rhl and Pqs circuits are also embedded in global post-transcriptional regulation. GacA/RsmY/RsmZ and PrrH connect signal-dependent regulation to biofilm, motility, phenazine, hydrogen cyanide, elastase and secretion-associated phenotypes [43,56].

### 2.2. Hierarchical and Reciprocal Regulation Among Las, Rhl, and Pqs

The classical PAO1 model places Las upstream of Rhl [58]. In this model, LasR, activated by N-(3-oxododecanoyl)-L-homoserine lactone (3-oxo-C12-HSL), promotes *rhlR* expression, and the Rhl system then controls C4-HSL-dependent outputs such as rhamnolipid production. The two systems interact: weak cross-activation can be detected, and maximal rhamnolipid production in a *lasI* mutant is not restored by signal supplementation alone. This hierarchy remains a useful laboratory starting model, but it is not a universal description of *P. aeruginosa* in host-associated environments [38,59].

Reciprocal regulation complicates a one-way Las-to-Rhl cascade. RhlR-mediated repression of PrrH relieves repression of *lasI* and phenazine genes, while PqsE and RhlR contribute to negative feedback on the *pqs* operon, and high concentrations of 4-hydroxy-2-heptylquinoline (HHQ) or PQS can down-regulate Pqs-associated transcription [37,43]. The direction and strength of regulation depend on the node, signal level and environmental condition. Network-level uncoupling further separates a regulator from the full phenotype set it controls. PqsR is closely tied to *pqsABCDE* activation, whereas PqsE controls multiple virulence and biofilm-associated regulons, including phenazine, hydrogen cyanide, rhamnolipid, lectin and cupE1 outputs [52,60]. PqsR antagonism may leave PqsE–RhlR outputs intact.

### 2.3. Strain Variation, lasR Deficiency, and Network Rewiring

Chronic infections can progressively remodel the QS network. Clinical cystic fibrosis data indicate that loss of Las-associated signaling may precede complete loss of Rhl-associated signaling, allowing Rhl-linked functions to persist after Las activity declines [7,59]. This pattern reflects a shift in pathway dependence rather than a global loss of quorum sensing. *lasR* deficiency does not produce a single predictable phenotype. Among 94 clinical isolates, two isolates carrying the same *lasR* truncating mutation differed in rhamnolipid, pyocyanin, protease, exotoxin A and biofilm phenotypes, and complementation restored individual outputs to different degrees [61]. Clinical-isolate transcriptomic analyses also show that *lasR*-deficient strains can converge on biofilm-adapted expression programs, indicating that genetic background and infection history shape the rewired state [62].

The cystic fibrosis isolate E90 provides a detailed example of Las-independent Rhl regulation. E90 produced high C4-HSL but only trace 3-oxo-C12-HSL, and RhlR, not LasR, was required for cytotoxicity in a three-dimensional airway epithelial model; the RhlR regulon also differed from the canonical PAO1 pattern [5]. In a PAO1 abscess model, residual virulence persisted after LasR or RhlR inactivation, indicating that alternative regulators, metabolic pathways and strain-specific circuits can maintain pathogenic phenotypes when the canonical QS node is lost [63].

### 2.4. Environmental, Host, and Infection-Stage Dependence

Nutrient conditions can reverse the apparent hierarchy. Under phosphate limitation, PhoB directly activated *rhlR*, and the transcriptional profile of a *lasR* mutant became much closer to wild-type PAO1 than under phosphate-replete conditions. Pyocyanin, elastase and rhamnolipid production remained high or became LasR-independent, whereas RhlR and PhoB remained necessary for the measured outputs. This places RhlR above LasR under defined infection-relevant stress conditions and shows why rich-medium network diagrams should not be treated as fixed properties of the species [34].

Iron availability provides another environmental control. Iron starvation induced the Pqs system, whereas excess iron reduced *pqsA* expression and could bind PQS or otherwise diminish its activity. PQS also drives iron-starvation responses, creating feedback between signal production and metal homeostasis. The resulting phenotype is shaped by bacterial density and by the chemical environment in which the signal accumulates [40].

Host-associated conditions add further selection. Chronic cystic fibrosis infection favors isolates with altered Las and Rhl function, whereas airway epithelial models show that epithelial injury can be LasR-dependent in PAO1 but RhlR-dependent in the clinical isolate E90 [5].

### 2.5. Implications of Quorum-Sensing Plasticity for Anti-Virulence Therapy

Network evidence argues against a universal ranking in which LasR is always the preferred therapeutic target. Target choice depends on the active signal system, isolate background, nutrient and metal conditions, and infection stage. Las-independent Rhl activity, PqsE-dependent RhlR outputs and PqsR-selective effects represent different target classes [26,34,52]. PQS-associated physical effects form a separate QS-adjacent category [39]. A biomarker-guided strategy based on isolate-specific QS profiles, including Las/Rhl/Pqs pathway activity, may eventually assist therapeutic selection; however, clinically validated predictive markers for QS-targeted therapy are currently unavailable.

## 3. QS-Associated Infection Phenotypes: Biofilm Formation, Virulence, and Antibiotic Tolerance

### 3.1. QS Regulation of Initial Attachment and Microcolony Formation

QS does not control initial attachment through a single matrix pathway. In PAO1, disruption of the GacA-RsmY/RsmZ layer reduced C4-HSL and several exoproducts but increased early biofilm development and swarming. Conversely, increased expression of PrrH, an Rhl-linked regulatory RNA locus, reduced early biofilm formation while increasing motility-associated phenotypes [43,56]. PqsE–RhlR biology also links downstream QS regulation to biofilm-associated community organization. Genetic studies show that PqsE-dependent RhlR activity can influence biofilm development and virulence-associated community phenotypes, even when canonical RhlI–C4-HSL signaling is insufficient to explain the output [36,54]. A regulatory role for the PqsE–RhlR axis should not be treated as direct evidence of initial attachment on a clinical surface. Clinical isolates also vary in their ability to form static biofilms. In a collection of 94 isolates, 11 isolates were biofilm-deficient, but the absence of protease did not reliably predict the biofilm phenotype. Defects in a single QS-regulated exoproduct cannot serve as a universal surrogate for impaired attachment or microcolony formation [61].

### 3.2. Biofilm Maturation, Matrix Production, and Architecture

Psl is regulated by QS at several levels. In stationary-phase PAO1 cultures, LasR contributed to *psl* transcription, whereas RhlR increased Psl production through translational or post-transcriptional control. In a double synthase mutant, both 3-oxo-C12-HSL and C4-HSL were required to restore Psl to wild-type levels. Although the study used 3-(N-morpholino)propanesulfonic acid (MOPS)-buffered lysogeny broth (LB) and does not establish the same degree of regulation in every clinical isolate, it documents distinct Las- and Rhl-mediated mechanisms controlling the same matrix component [64].

PqsE and related downstream pathways also shape biofilm-associated community structure. Network-uncoupling and transcriptomic studies link PqsE to cupE1, lectin, rhamnolipid, phenazine and hydrogen cyanide programs associated with biofilm development [52,60]. Genetic studies connect the PqsE–RhlR axis to biofilm-associated and virulence-related fitness [36,54]. The PqsE–RhlR axis remains a candidate downstream target, but the reviewed evidence does not include a clinically usable inhibitor.

A 72 h PA14 study separated biofilm biomass from viable-cell burden. *lasI*- and *lasR*-deficient mutants eventually produced more crystal-violet-stained biomass than the wild type yet yielded fewer recoverable viable cells. Excess biomass was associated with autolysis, dead-cell material and altered matrix production. Increased staining cannot be interpreted as increased bacterial load, greater viable-cell density or necessarily more severe infection [17].

### 3.3. Biofilm Dispersion and Outer-Membrane-Vesicle-Related Effects

Dispersion is a distinct developmental state, not a synonym for biofilm maturation or eradication. In PA14 flow-cell biofilms, PQS and outer membrane vesicle production peak during dispersion [39]. Mutants defective in PQS production or PqsR showed impaired central void formation and reduced dispersion, whereas exogenous PQS rescued dispersion in the *pqsR* mutant. The *pqsE* mutant dispersed normally, indicating that this process reflected the physical effect of PQS-induced vesicle formation rather than the complete PqsE-dependent transcriptional program. Purified vesicles exhibit protease, lipase and DNase activities that can degrade matrix components [65,66].

PQS-outer membrane vesicle (OMV) signaling can release viable, motile cells from mature communities and shift the relationship between persistence and dissemination. Dispersion might increase antibiotic access if paired with effective killing, but it may also expose tissue to a more acute or mobile population. Because only limited amounts of biofilm-derived material could be recovered, the matrix-degradation assays used vesicles from planktonic cultures; the extent of comparable activity in mammalian infections remains uncertain [66,67,68].

### 3.4. Regulation of Virulence Factors and Secretion-Associated Phenotypes

In planktonic PAO1 cultures, the Las and Rhl systems regulate a broad set of secreted or surface-active virulence traits. LasR controls elastase, LasA protease, alkaline protease and exotoxin A-associated gene expression, whereas RhlR controls rhamnolipid biosynthesis through *rhlAB* expression. The foundational study in planktonic PAO1 cultures defines regulatory relationships but does not establish host injury or a universal in vivo output profile [69].

QS-associated regulators modulate motility and surface persistence in a context-dependent manner. GacA–Rsm and PrrH perturbations illustrate different responses in laboratory systems [43,56]. These findings do not establish that an intervention reducing motility or biofilm biomass will prevent dissemination or improve clinical outcomes. In cystic fibrosis isolates, Rhl-related outputs may persist despite attenuation of Las-associated signaling [7,38,59].

### 3.5. Motility, Colonization, Tissue Spread, and Chronic Infection

QS-associated regulators help coordinate the trade-off between motility and surface residence. GacA-Rsm mutants showed increased swarming and early biofilm development, whereas PrrH-locus overexpression increased swimming and swarming but reduced biofilm formation [43,56]. These apparently opposing effects are consistent with QS acting as a lifestyle regulator rather than a unidirectional biofilm switch. In host-associated settings, regulatory rewiring means that colonization and tissue-spread phenotypes cannot be inferred from laboratory regulatory hierarchies alone. Clinical cystic fibrosis data show the persistence of Rhl-associated signaling and virulence outputs after Las activity declines [38,59].

### 3.6. Antibiotic Tolerance, Persister States, and Biofilm Microenvironments

Antibiotic resistance and tolerance should be distinguished [70]. Resistance is an inherited reduction in antimicrobial susceptibility, reflected in many studies by an increased planktonic MIC. Tolerance is the ability of cells to survive transient antimicrobial exposure without a necessary change in MIC, in association with growth state, metabolic heterogeneity, matrix structure or stress adaptation. In PA14 colony biofilms, phenazine production increased survival against several antibiotics without changing planktonic MICs, providing a direct example of biofilm-associated tolerance rather than inherited resistance [9]. QS-linked pathways can contribute to persister formation without making the organism intrinsically resistant. PqsR inhibition reduced persister-associated phenotypes and limited relapse in preclinical infection models [10]. The result implicates PqsR in persistence, not in all forms of biofilm tolerance.

Phenazines illustrate a downstream, QS-adjacent route to metabolically conditional tolerance. In PA14 colony biofilms, phenazine production protected cells from ciprofloxacin, tobramycin and carbenicillin under glucose-fed conditions but increased susceptibility to colistin. This effect depended on oxygen limitation and central metabolism rather than a simple increase in matrix or efflux. The study did not directly manipulate PQS, Las, Rhl, PqsR or PqsE and cannot establish PQS as a general determinant of antibiotic tolerance [9].

Direct QS disruption likewise did not uniformly sensitize biofilms to antibiotics. In the 72 h PA14 study, *lasI* and *lasR* mutants showed no consistent increase in susceptibility to chloramphenicol, tetracycline or carbenicillin relative to the wild type; the *lasR* mutant was more susceptible only under a single high-dose carbenicillin condition. This finding again separates biofilm biomass and viable-cell burden from antibiotic tolerance [17].

## 4. Quorum-Quenching and Broader Quorum-Sensing Interference Strategies

### 4.1. Conceptual Classification of Quorum-Quenching Mechanisms

**Figure 2:**
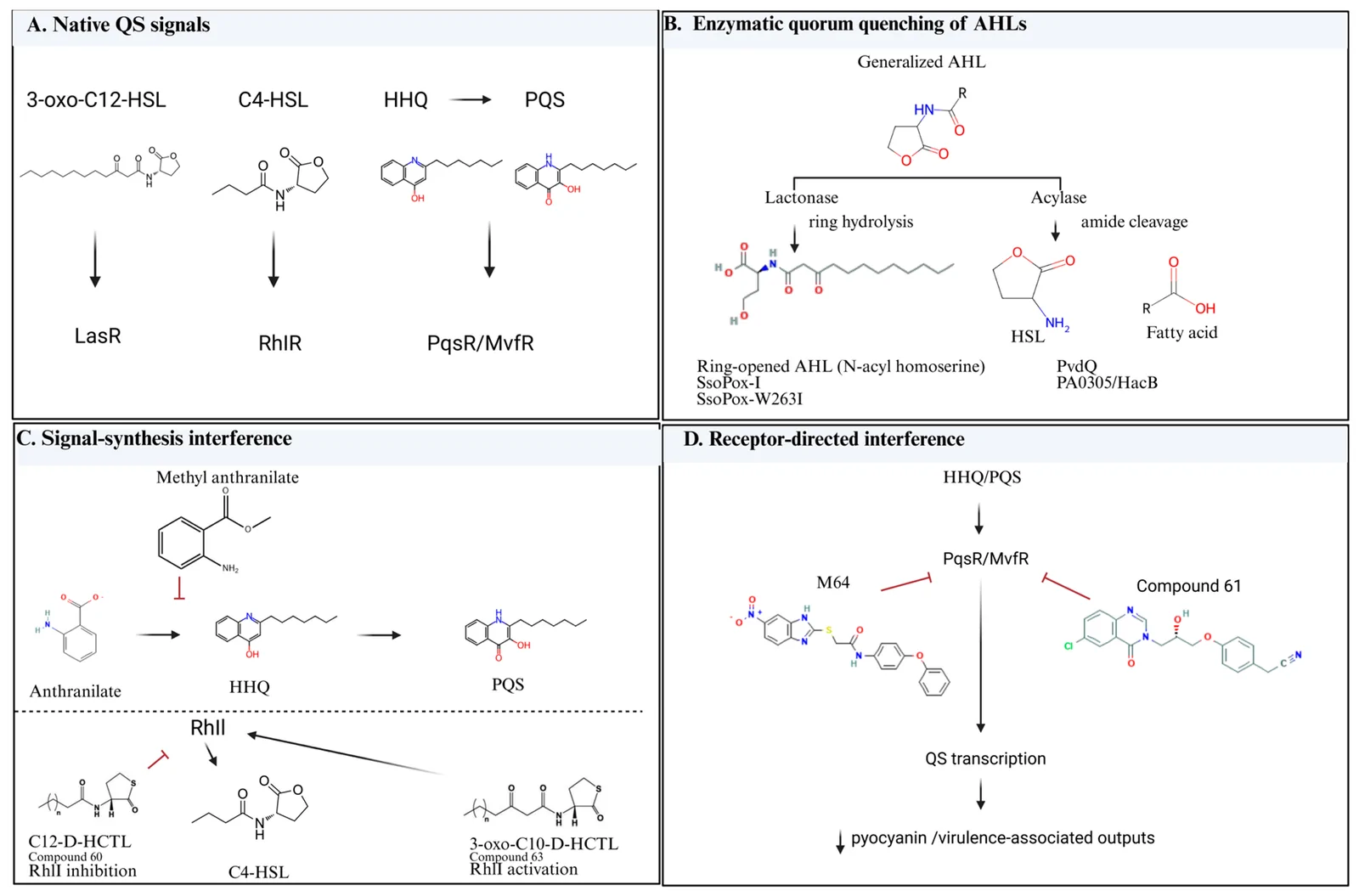
Chemical signals, enzymatic quorum quenching and representative synthesis- and receptor-directed QS interference in *P. aeruginosa*. (**A**) Native AHLs (3-oxo-C12-HSL and C4-HSL) and alkylquinolones (HHQ and PQS). (**B**) Lactonases hydrolyze the AHL lactone ring to a ring-opened product; acylases cleave the amide bond to release homoserine lactone and the corresponding fatty acid. (**C**) Representative signal-synthesis interference; synthase inhibitors and activators are not interchangeable. (**D**) PqsR-directed antagonists act at the receptor rather than chemically degrading the signal. Solid arrows depict the indicated chemical transformations or signaling relationships, and red T-bars indicate inhibition. In the chemical structures, oxygen, nitrogen, sulfur, and chlorine atoms are represented in red, blue, yellow, and green, respectively, while carbon atoms are shown in gray or black. Representative reaction classes and intervention examples are supported by the original biochemical and chemical studies [10,24,25,26,42,73].

Mechanism assignment precedes efficacy grading. Canonical QQ requires direct evidence that a signal is degraded, chemically modified, or sequestered. Signal-synthesis inhibition, LasR/RhlR/PqsR antagonism, PqsE–RhlR interference, and QS-adjacent effects are distinct mechanisms. Reporter activity or a QS-regulated phenotype alone is inadequate to attribute a direct molecular target [23,24,71,72]. Figure 2 summarizes the native QS signals of *P. aeruginosa* and illustrates representative mechanisms of enzymatic quorum quenching, signal-synthesis interference, and PqsR-directed antagonism. Table 1 reports target-level evidence, model context and limitations without assigning a composite grade for therapeutic readiness.

**Table 1:** Targets, validation, models, and evidence limitations of representative quorum-sensing interventions in *Pseudomonas aeruginosa*.

Intervention/Agent	Target/Mechanism	Direct Evidence and Verified Quantitative Findings	Main Finding	Evidence Context	Interpretation/Limitation	Ref.
PvdQ	AHL acylase; 3-oxo-C12-HSL	AHL amide-bond hydrolysis; signal depletion and growth controls	Reduced QS outputs in mouse pulmonary infection models	Mechanism + mammalian infection	Target-site exposure and repeated-dose effects not established	[20,23]
PA0305/HacB	AHL acylase; Las-associated long-chain AHLs	Purified-enzyme hydrolysis; kcat/km 1.4 × 10^3^ M^−1^ s^−1^ (C8-HSL), 7.8 × 10^4^ M^−1^ s^−1^ (3-oxo-C12-HSL)	Signal turnover and genetic virulence phenotypes	Biochemical + genetic	Genetic phenotypes do not establish the therapeutic efficacy of an administered enzyme	[24]
SsoPox-I	AHL lactonase	AHL ring hydrolysis; QS reduction	Acute rat pneumonia: 75% untreated vs. 20% immediate-treatment mortality	Enzymatic + mammalian infection	Acute timing-dependent effect; bacterial burden not consistently reduced	[21]
SsoPox-W263I	Engineered AHL lactonase	AHL hydrolysis and clinical-isolate QS-phenotype assays	51 diabetic-foot clinical isolates; 0.5 mg/mL in vitro test concentration (not an administered dose)	Enzymatic + clinical-isolate laboratory models	Not equivalent to SsoPox-I; no demonstrated human treatment efficacy	[1,2]
AidC and QQ-2	AHLs (AidC) and externally supplied AI-2 (QQ-2); interspecies signaling context	Combined extracellular quenching during PAO1 in vitro AI-2 challenge	Reduced protease, elastase, pyocyanin and biofilm-associated phenotypes	In vitro signaling/phenotype	*P. aeruginosa* lacks endogenous luxS-mediated AI-2 synthesis; exogenous-signal assay, not mammalian efficacy	[74]
Methyl anthranilate	Anthranilate-derived alkylquinolone synthesis	Precursor-competition/interference with PQS synthesis	Reduced PQS-associated output in bacterial assays	Cellular biochemical/pathway evidence	Direct isolated-enzyme inhibitory potency and efficacy in vivo have not been established	[42]
RhlI analogues (Compounds **60** and **63**)	RhlI signal synthase	Purified RhlI modulation: inhibitor 60 IC50 11 ± 1 µM; activator 63 EC50 57 ± 23 µM	Opposite modulation modes; not both inhibitors	Purified-enzyme biochemical	Efficacy in infection models has not been established; pharmacological selectivity in cells remains unresolved	[73]
M64	PqsR/MvfR	QS-pathway suppression, quinolone readouts and infection-model target evidence	Improved survival in murine infection models	Chemical + mammalian infection	Animal-model dependence; exposure and clinical translation unresolved	[10]
Compound **61**	PqsR/MvfR	Binding Kd 10 nM; receptor co-crystal resolution 2.65 Å; reporter IC50 1 µM	Reduced alkylquinolone/QS readouts	Structural + cellular	Efficacy in vivo should not be inferred from binding potency	[26]
Compound **40**	PqsR/MvfR	Reported PAO1-L IC50 0.25 ± 0.12 µM; PA14 potency differs	PqsR pathway/reporting inhibition	Cellular pharmacology	Separate original chemical series; no universally established outcome in vivo	[25]
PqsE–RhlR axis	Direct protein–protein interface	Genetic, protein-interaction, structural, and DNA-binding studies	Validated regulatory dependency of RhlR-associated virulence	Target biology (non-drug)	Does not establish a clinically active PqsE–RhlR inhibitor	[27,29,36,37]

Table 1 note. The entries report the strongest attributable target- or signal-level evidence for each representative agent. Genetic target validation is not equivalent to pharmacological infection efficacy. IC50, EC50 and Kd values refer to the corresponding experimental assay and should not be compared across assay systems. AI-2 quenching in PAO1 experiments concerns externally supplied AI-2 [74]. Abbreviations: AHL, N-acyl homoserine lactone; AI-2, autoinducer-2; c-di-GMP, cyclic diguanylate; CFTR, cystic fibrosis transmembrane conductance regulator; HDMF, 4-hydroxy-2,5-dimethyl-3(2H)-furanone; HPLC, high-performance liquid chromatography; HHQ, 4-hydroxy-2-heptylquinoline; ITC, isothermal titration calorimetry; MIC, minimum inhibitory concentration; PQS, Pseudomonas quinolone signal; qPCR, quantitative PCR; QQ, quorum quenching; QS, quorum sensing.

Mechanistic class also determines how medical effects should be interpreted. A signal-degrading enzyme may reduce quorum activity without lowering bacterial numbers, whereas a receptor antagonist may suppress only a subset of outputs. A delivery formulation may introduce antibiotic, membrane-disrupting, anti-adhesive, or matrix-active effects that cannot be attributed to QS interference without appropriate controls. Examples that pair target or signal measurements with explicit growth controls and, where possible, host or infection outcomes include PvdQ and SsoPox enzymes, the M64 PqsR inhibitor, and mechanistic studies of the PqsE–RhlR interface [1,10,20,21].

### 4.2. Direct Signal Degradation, Modification, and Sequestration

Enzymatic signal degradation is the most direct form of canonical quorum quenching [75,76]. PvdQ is an acylase that cleaves the amide bond of 3-oxo-C12-HSL, and its activity was linked directly to signal depletion, growth-independent virulence reduction and host protection in *Caenorhabditis elegans* [23]. PA0305, termed HacB in the relevant study, was characterized using purified-enzyme kinetic assays and high-performance liquid chromatography (HPLC) to identify reaction products, establishing hydrolysis of long-chain AHLs rather than a nonspecific toxic effect [24]. Both studies used genetic loss- and gain-of-function approaches and reported no material growth defect under the tested conditions. PA0305 lacked activity against C4-HSL and PQS, whereas the periplasmic location of PvdQ may restrict access to extracellular or biofilm-associated signal pools.

Engineered quorum-quenching enzymes extend this principle by improving catalytic or biochemical properties [77]. Pulmonary studies of SsoPox-I and PvdQ extend enzymatic quorum quenching into host models but also reveal timing, burden and delivery constraints [20,21]. SsoPox-W263I was evaluated across diabetic-foot and cystic fibrosis isolate collections, with growth controls and assessments of selected protease, pyocyanin, elastase, and biofilm phenotypes [1,2,44]. SsoPox-mediated signal disruption has also been reported to alter CRISPR-Cas expression in model and clinical *P. aeruginosa* strains, illustrating the wider regulatory consequences of enzymatic QS interference rather than therapeutic efficacy per se [78]. Clinical-isolate studies broaden the evidence beyond laboratory strains, yet responses remain heterogeneous across isolates and tissues.

Substrate specificity is a central target-selection variable, not a biochemical detail. SsoPox-W263I and GcL produced comparable depletion of the selected AHLs but had divergent effects on PQS, the proteome, biofilm, antibiotic sensitivity and amoeba pathogenicity; only SsoPox-W263I reduced pathogenicity in the amoeba model [79]. In the cystic fibrosis isolate collection, individual traits were inhibited or stimulated depending on the lactonase and isolate [44]. SsoPox-W263I also potentiated antibiotic and phage activity in a panel of clinical and phage-resistant strains; one isolate remained refractory in the host model, and AHL depletion was not directly quantified during treatment [2]. A dual-enzyme study combined an AHL lactonase with an autoinducer-2 (AI-2)-quenching enzyme and reduced virulence factors under an AI-2 challenge. This work was limited to PAO1 in vitro and did not address PQS [74].

The role of AI-2 requires a distinction between production and interspecies sensing. *P. aeruginosa* lacks the canonical luxS gene and does not ordinarily synthesize AI-2, but it can respond to AI-2 produced by neighboring bacteria. Oropharyngeal-flora-derived or experimentally supplied AI-2 altered subsets of *P. aeruginosa* virulence-associated gene expression in experimental systems, and the chemoreceptors PctA and TlpQ have been shown to bind AI-2 and mediate chemotactic and biofilm-associated responses [80,81]. Accordingly, the AidC/QQ-2 co-treatment results after an exogenous AI-2 challenge should be interpreted as interference with interspecies signaling rather than blockade of a native AI-2 biosynthetic pathway in *P. aeruginosa*; these in vitro findings do not establish efficacy against polymicrobial infection [74].

Direct signal sequestration offers a non-enzymatic form of canonical QQ. A structure-guided bifunctional aptamer was reported to recognize the AHL signals 3-oxo-C12-HSL and C4-HSL; reductions in rhamnolipid, pyocyanin, and biofilm readouts were observed in experimental systems rather than clinical infection studies [82]. Aptamers sequester signals without chemical degradation, unlike lactonases and acylases. Binding specificity, biological-fluid stability and infection-site exposure require further validation.

Signal degradation does not invariably inhibit biofilms. HodC-mediated PQS degradation increased newly formed and established biofilm biomass in a PA14 lasB::Tn background under defined experimental conditions, with increased iron availability proposed as a contributing mechanism [83]. This strain- and assay-specific counterexample demonstrates why direct chemical evidence of signal depletion must be evaluated alongside viable biomass and infection-relevant outcomes, rather than interpreted as an automatic therapeutic benefit.

### 4.3. Inhibition of Signal Synthesis

Direct inhibition of signal synthesis is less extensively studied than enzymatic degradation. Methyl anthranilate interfered with anthranilate-dependent PQS production in PAO1, as shown by radiolabeled precursor tracing and direct measurement of PQS by extraction and densitometry. The reduction in PQS was accompanied by a dose-dependent decrease in elastase, whereas bacterial growth was reported to be unaffected at the tested concentrations [42]. AHL synthases are less developed as pharmacological targets than signal-degrading enzymes or receptor antagonists. Structure–activity studies with purified RhlI showed that selected long-chain acyl-D-homoserine lactones and acyl-D-homocysteine thiolactones inhibit the synthase, whereas some 3-oxoacyl analogs can activate it; the reported effects were measured with biochemical assays and examined by mechanistic modeling [73]. Additional screening studies identified natural-product-derived compounds that inhibit AHL synthase activity in biochemical or cell-based assays, including salicylic acid, tannic acid and trans-cinnamaldehyde [84]. Structure–function studies of RhlR ligands show that chemical modification of the native AHL scaffold can produce potent receptor modulators [85]. Synthase and receptor modulation act at distinct mechanistic levels; these findings do not establish a validated dual LuxI/LuxR strategy. These findings provide separate chemical starting points that still require native-pathogen target engagement, permeability, growth and infection-model validation.

### 4.4. Receptor Antagonism Targeting LasR, RhlR, and PqsR (MvfR)

When evaluating receptor antagonists, direct target engagement is more informative than cell damage or reductions in downstream phenotypes. Receptor-selective reporters, ligand competition, direct binding, structural measurements, covalent chemistry controls and growth-matched assays provide stronger evidence than quantitative PCR (qPCR) or biofilm reduction alone [71,72,86,87]. Crystal structures of the LasR and PqsR ligand-binding domains define the pockets used to assess antagonist engagement [88,89], while systematic structure–activity relationship (SAR) studies of AHL analogs have mapped structural requirements for agonist versus antagonist activity [90,91]. RhlR studies now include the same types of receptor-focused measurements. Sulfonyl homoserine lactone probes inhibit RhlR and dock into its ligand-binding site [92], while PqsE binding to RhlR fine-tunes transcription in a promoter-specific manner, marking the PqsE–RhlR interface as a downstream regulatory target [37].

RhlR perturbation also shows that receptor inhibition does not map onto a simple linear hierarchy. Selective RhlR antagonists have been validated as chemical probes for receptor modulation [72], whereas related chemical-genetic studies show that perturbing RhlR can produce inverse effects on pyocyanin and rhamnolipid and alter Pqs cross-regulation [86]. Environment-specific experiments show that the relative importance of Las, Rhl and Pqs changes under phosphate- and iron-limited conditions [87]. RhlR modulation can redirect network activity rather than uniformly switch off all QS-dependent phenotypes.

PqsR is one of the most extensively studied receptor targets in the *P. aeruginosa* medicinal-chemistry literature. M64 was bound to the MvfR co-inducer-binding domain, reduced promoter occupancy and alkylquinolone/pyocyanin outputs, limited persister-associated phenotypes, and was associated with better outcomes in acute and persistent murine infection models, including multi-drug-resistant isolates [10]. Compound **61** had binding and structural validation and potentiated ciprofloxacin during an early treatment window in pre-established biofilms and in *Galleria mellonella*; monotherapy was weak, and later or clinical-isolate effects were less consistent [25,26]. Compound **40** also reduced PqsR-associated signals but did not increase ciprofloxacin killing in a mature biofilm assay and slightly increased biofilm viability under one condition [25,26]. A study of a newer inhibitor series also found that permeability and efflux limited activity in native bacterial cells [93]. PqsR is a pathway-specific anti-virulence target; its inhibition does not imply suppression of all PqsE–RhlR or receptor-independent PQS effects.

Thiazole-based PqsR inverse agonists represent a chemical series distinct from M64. Optimization of this series yielded lead compounds **27** and **35** with single-digit nanomolar target potency, nanomolar effects on pyocyanin and alkylquinolone biosynthesis, and pharmacological profiling in vitro [94]. These data broaden chemical-series coverage but do not demonstrate equivalence to infection-model efficacy, human target-site exposure, or established clinical benefit. Comparison of receptor antagonism with signal-degrading QQ requires separate appraisal of mechanism, assay quality and infection-model relevance. Although SsoPox variants, PvdQ, and M64 provide some of the most complete examples linking target-level validation with infection-model outcomes, they should not be interpreted as representing the entire QS-interference landscape. Other chemical series, including additional PqsR antagonists, RhlR modulators, and signal-synthesis inhibitors, provide complementary mechanistic evidence but remain at earlier translational stages.

### 4.5. PqsE–RhlR and Downstream Regulatory-Network Interference

Targeting the PqsE–RhlR axis represents a distinct form of downstream quorum-sensing interference. Earlier genetic work showed that PqsE is required for several RhlR-dependent virulence outputs, even when canonical RhlI–C4-HSL signaling is insufficient to explain the phenotype [36,54]. Subsequent biochemical, transcriptomic, interactomic and structural studies established a direct PqsE–RhlR protein–protein interaction that increases RhlR DNA binding and promoter activity, while demonstrating that this regulatory interaction is separable from PqsE catalytic activity [27,53,57]. The earlier concept of a PqsE-derived alternative ligand should be treated as a historical model rather than an established chemical mechanism.

Structure–function work separates biological target validation from pharmacological tractability. PqsE variants that disrupt the interaction with RhlR impair RhlR-dependent virulence programs, whereas an active-site binder such as BB584 does not by itself disrupt the PqsE–RhlR interface [29]. Together with studies directly characterizing the complex and its interactions [27,53,57], these data establish the PqsE–RhlR interface as a biological target, while the reviewed literature does not establish a mature class of small-molecule inhibitors of this interface.

Other downstream studies map regulatory nodes with therapeutic relevance without demonstrating pharmacological quorum quenching. Genetic uncoupling of HHQ, PQS, 2-heptyl-4-hydroxyquinoline N-oxide (HQNO) and PqsE showed that PqsE controls multiple virulence and biofilm-associated regulons, whereas PQS also affects iron-starvation responses, and HQNO is not a QS signal [52]. Under phosphate limitation, PhoB can place RhlR at the top of the hierarchy and bypass LasR [34]. The PrrH small-RNA cascade and GacA-dependent RsmY/RsmZ regulation connect QS to virulence and biofilm control, whereas the c-di-GMP study is a related but QS-adjacent analysis of lifestyle regulation rather than a quorum-quenching intervention [43,56,95]. These studies define candidate regulatory targets rather than established pharmacological interventions.

### 4.6. Target Selection, Network Redundancy, and Strain-Dependent Limitations

Target selection should follow the active regulatory branch rather than a universal Las-to-Rhl hierarchy. Clinical isolates can lose Las-associated signaling while retaining RhlR-dependent outputs, and the phenotype of *lasR*-defective isolates varies with genetic background and biofilm adaptation [61,63]. Environmental conditions can shift the hierarchy, as shown by RhlR dominance under phosphate limitation [34]. LasR-directed therapy may leave clinically relevant Rhl or PqsE–RhlR activity intact in some isolates.

Target choice also depends on substrate specificity, environmental state, permeability and delivery. Lactonase studies found that similar nominal AHL depletion can produce different downstream states [44,79]. MexAB-OprM markedly reduces the apparent potency of AHL-derived QS inhibitors, so heterologous reporter activity may overestimate activity in native *P. aeruginosa* [12]. PqsR inhibitors likewise encounter uptake and efflux barriers [93]. Delivery further modifies exposure: formulation charge and mucus or matrix interactions can increase penetration or, under some conditions, promote biofilm accumulation [14,15]. Candidate agents should be tested against the relevant target in native *P. aeruginosa* backgrounds with matched growth controls, permeability assessment, clinical-isolate diversity and infection-relevant environmental conditions. Multi-node strategies are most defensible when bypass has been observed and interaction is measured directly [96].

## 5. Anti-Virulence Effects, Host Protection, and Medical Applications

### 5.1. Biofilm and Virulence Attenuation

QS interference can attenuate *Pseudomonas aeruginosa* biofilm development and virulence, but effect size and direction depend on target, strain, biofilm stage and treatment context. Direct signal-degrading enzymes and receptor antagonists can reduce selected early-biofilm or virulence phenotypes when target engagement is measured and matched growth controls are included [44,71,97]. Comparable signal depletion does not guarantee comparable downstream phenotypes, and clinical isolates may show inhibition, no response or stimulation of individual traits [44,79]. QS-adjacent compounds, including antimicrobial peptides and host-active or pleiotropic small molecules, can also reduce selected biofilm or virulence endpoints, but without direct target attribution they remain QS-adjacent rather than canonical quorum quenchers [98,99,100,101].

Activity against established biofilms is less consistent than prevention of early biofilm development. PqsR antagonist compound **40** suppressed HHQ and PQS but did not increase ciprofloxacin killing of mature biofilms and slightly increased biofilm viability under one condition [25]. Nanoparticle-based co-delivery can increase penetration and antibiotic efficacy. Squalenyl hydrogen sulfate nanoparticles carrying a PqsR inhibitor and tobramycin penetrated mucus and 24-h biofilms, achieving eradication at a lower tobramycin exposure than free antibiotics. [14]; 3-amino-7-chloro-2-nonylquinazolin-4(3H)-one (ACNQ)–ciprofloxacin alginate nanoparticles increased killing in mature biofilms and ex vivo skin models [102]. These experiments support the potential of combination delivery but do not establish QQ monotherapy efficacy against mature biofilms. Signal suppression, biomass reduction, dispersion, viable-cell killing and eradication are distinct outcomes.

### 5.2. Antibiotic Sensitization and Combination Therapy

Antibiotic sensitization is a conditional rather than intrinsic consequence of QS inhibition [102]. SsoPox-W263I increased ciprofloxacin or phage activity against several clinical isolates, whereas compound **61** produced only time-dependent ciprofloxacin potentiation and weaker effects in some clinical isolates [2,26]. LasR-inhibitor–ciprofloxacin conjugates and other anti-virulence compounds provide additional combination evidence. Direct receptor attribution and the contribution of the antibiotic component differ among platforms [103,104]. Hybrid systems can confound interpretation: antibiotics, membrane disruption, matrix degradation, anti-adhesive materials and photothermal effects may independently contribute to cell death [105,106]. Combination studies should compare QS-directed agents alone, antibiotics alone, their combinations, and appropriate inactive-agent or target-deficient controls.

### 5.3. Host-Protective Effects

A rationale for anti-virulence therapy is that host protection can occur without concurrent bacterial clearance. SsoPox-I reduced mortality and lung pathology in acute rat pneumonia with little change in lung bacterial burden, whereas PvdQ reduced inflammatory and histological injury with model-dependent effects on bacterial counts and survival [20,21]. M64 was associated with better outcomes in pulmonary, burn, macrophage and persistence models [10], and palmitoleic acid reduced epithelial apoptosis, neutrophilic inflammation and lung pathology without reducing bacterial burden in the tested prophylactic model [8]. Bacterial burden, tissue pathology, inflammation, cell death and survival should be treated as separate endpoints [107].

Host-cell studies also separate pathway modulation from bacterial clearance. LasR-associated bacterial exoproducts impair cystic fibrosis transmembrane conductance regulator (CFTR) maturation, localization and chloride-channel function in airway epithelial models, whereas 4-hydroxy-2,5-dimethyl-3(2H)-furanone (HDMF) preserves CFTR function without affecting bacterial growth at the tested concentration [108]. This effect cannot be attributed to a single QS target because HDMF is not LasR-specific. Farnesol and tyrosol reduced selected epithelial inflammatory and adherence phenotypes, but responses varied between strains and host-cell systems [100]. The 3-oxo-C12-HSL signal can accelerate apoptosis in macrophages and neutrophils; this finding is mechanistically distinct from evidence that QQ alters neutrophil recruitment [109]. Evidence that QQ directly reduces host oxidative injury is sparse; PQS–iron interactions define a bacterial mechanism but not host reactive oxygen species (ROS) reduction [40]. Improved epithelial repair-related outcomes after QS inhibition were reported in an airway epithelial study; the evidence remains preclinical and host-cell-based [110]. A recent review adds host-context discussion but no new intervention efficacy data [111].

### 5.4. Medical Applications and Delivery Strategies

Human evidence for QS-directed benefit remains exploratory. A multicenter randomized, placebo-controlled trial enrolled 92 mechanically ventilated patients colonized with *P. aeruginosa* and compared azithromycin (300 mg/day) with a placebo. In the per-protocol population (43 azithromycin-treated and 42 placebo-treated patients), *P. aeruginosa* ventilator-associated pneumonia occurred in 2/43 (4.7%) versus 6/42 (14.3%), respectively; the difference in the overall analysis was not statistically significant (*p* = 0.156). In an exploratory subgroup persistently colonized by isolates producing high levels of rhamnolipids, pneumonia occurred in 1/5 (20%) azithromycin-treated patients versus 5/5 (100%) placebo-treated patients (*p* = 0.048) [112]. Despite this nominal subgroup *p* value, the comparison involved only ten patients, yielding an extremely imprecise effect estimate; selection of a subgroup based on a bacterial phenotype, multiplicity and the absence of a statistically significant overall treatment effect precludes a confirmatory efficacy inference. Moreover, azithromycin has antimicrobial and immunomodulatory effects, so the clinical outcome cannot be specifically attributed to quorum-sensing inhibition.

A randomized pilot trial of garlic capsules in cystic fibrosis enrolled 34 participants, with 26 included in the per-protocol analysis after eight weeks of garlic or placebo capsules (656 mg/day). Between-group differences in FEV1 change and other clinical outcomes were not statistically significant, and the effect, if any, could not be attributed directly to QS blockade [113]. These human investigations justify continued clinical study but not a claim of validated QQ-mediated clinical benefit. Future trials should measure QS target-engagement biomarkers alongside clinical endpoints relevant to patients.

Within the reviewed literature, pulmonary infection is among the most extensively studied application settings. Local delivery can address high bacterial density, mucus barriers and QS-regulated injuries. SsoPox-I and PvdQ have shown activity in acute pulmonary challenge models, whereas niclosamide formulations and inhalable AHL-acylase powders represent quorum-sensing inhibitor (QSI)-adjacent and canonical-QQ pulmonary delivery approaches, respectively [114,115,116]. In cystic fibrosis, clinical adaptation and persistence of Rhl-associated signaling after Las decline argue against reliance on a single universal QS target [108,117]. The reviewed literature provides little direct evidence for QS-targeted treatment of chronic obstructive pulmonary disease or ventilator-associated pneumonia.

**Figure 3:**
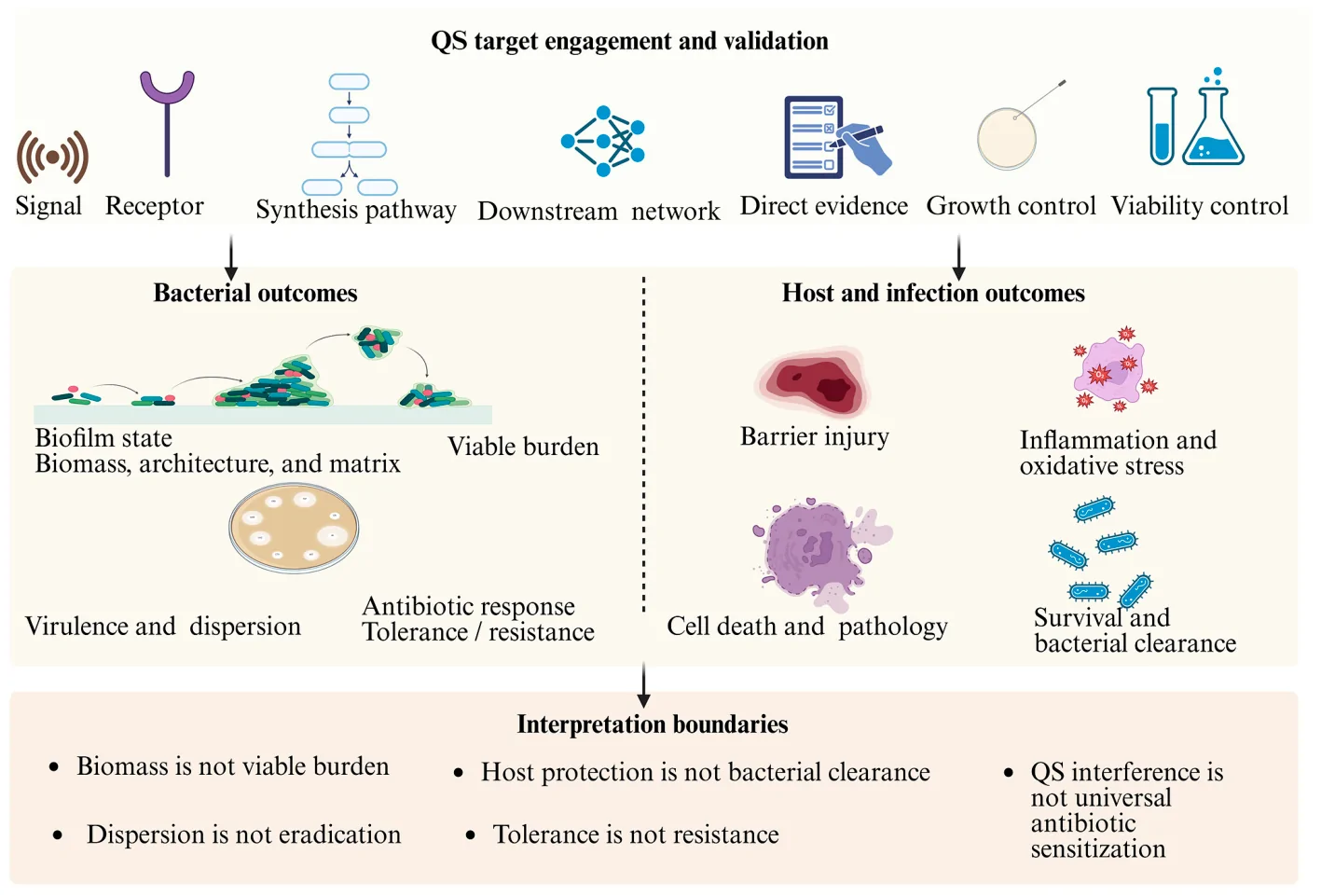
From QS target engagement to bacterial and host outcomes. The upper panel summarizes intervention points (QS signals, receptors, signal-synthesis pathways and downstream regulation) and the requirement for direct mechanistic evidence with matched bacterial growth and viability controls. Bacterial outcomes include biofilm biomass, architecture, viable burden, virulence, dispersion and antibiotic response. Host and infection outcomes include epithelial injury, inflammation, cell death, tissue pathology, survival and bacterial clearance. The dashed separator distinguishes bacterial from host endpoints. Arrows indicate the sequence of evidence assessment, not established causal effects of every QS intervention. Biofilm biomass is distinct from viable burden, dispersion from eradication, tolerance from resistance, and host protection from bacterial clearance.

Delivery technology can alter efficacy. Mucin adsorption reduces free-QSI activity, whereas appropriately designed nanoparticles can increase mucus and biofilm penetration [14]. Cationic or chitosan-coated formulations can become trapped within biofilms and, under certain conditions, increase mature-biofilm density [15]. Protein corona formation may alter enzyme orientation, catalytic accessibility, and tissue distribution, meaning that in vitro catalytic activity may not predict activity in airway mucus or wound environments [47]. Carrier charge, matrix binding, enzyme stability and formulation-associated antibacterial effects should be evaluated alongside target engagement. Locally delivered QQ and QSI approaches remain preclinical adjunctive strategies rather than stand-alone antibacterial approaches (Figure 3, Table 2).

Local delivery presents challenges beyond formulation design. Hydrophobic agents may bind airway mucins, while nanoparticle protein coronas may change surface chemistry and distribution; charge- and matrix-dependent adsorption can reduce penetration. For enzyme-based therapies, activity and stability in airway secretions or wound fluid, substrate access, proteolysis, and immune responses to repeated dosing require direct measurement. Qualitative evidence of deposition or short-term efficacy does not establish sustained target-site activity [14,15,47].

**Table 2:** Representative infection, clinical-isolate, ex vivo, prevention, and formulation studies: model-specific findings.

Evidence Setting	Intervention	Model, Concentration/Dose, and Timing	Quantitative Findings	Interpretation/Primary Limitation	Ref.
Animal infection	SsoPox-I	PAO1 acute rat pneumonia; intratracheal 250 µL at 1 mg/mL (=0.25 mg SsoPox-I per rat), once immediately or 3 h after infection; *n* = 20/group	Mortality: untreated 15/20 (75%); immediate 4/20 (20%, *p* = 0.0001); delayed 10/20 (50%, not significant vs. control)	Host survival improved with immediate dosing; no consistent lung-CFU reduction	[21]
Animal infection	PvdQ	Mouse pulmonary agarose-bead infection; intranasal 25 or 250 ng/g body weight once daily (mass normalized to body weight, not concentration); lethal challenge 5 × 10^5^ CFU/lung; *n* = 6 for lethal survival comparison	25 ng/g: ≈5-fold reduction in burden (*p* = 0.0465); median survival 57 vs. 42 h (*p* = 0.004)	Acute model; greater doses do not automatically mean better exposure	[20]
Animal infection	M64	PA14 murine burn infection; IV 4 mg/kg from 6 h postinfection and then twice daily for 6 days; survival *n* = 30 untreated, *n* = 36 treated	Survival improvement *p* < 0.001; early CFU differences not significant through day 7; day-11 CFU *p* < 0.001	Direct preclinical host effect distinct from immediate bactericidal clearance	[10]
Animal infection	M64 (lung model)	PA14 murine pneumonia; IV 4 mg/kg at 2, 4, 8, 12 h and then twice daily through day 4; ≥10 animals/group	Survival benefit *p* < 0.05; lung HHQ reduced; early 14 h bacterial burden unchanged	Separate acute lung model and dosing schedule; distinct from the burn-infection experiment	[10]
Clinical-isolate laboratory	SsoPox-W263I	51 diabetic-foot clinical isolates; 0.5 mg/mL final enzyme concentration in vitro (no dose administered to animals or patients); virulence assays 24 h, biofilm assays 48 h	Reduced selected QS outputs with heterogeneous isolate responses	Clinical isolate origin does not establish efficacy in patients or animal infection models	[1,2]
In vitro/ex vivo	ACNQ + ciprofloxacin nanoparticles	Established biofilms, keratinocytes and 3D ex vivo skin; combination formulation	Enhanced biofilm clearance relative to comparator in tested systems	Quantitative values not tabulated because cross-condition data were not securely comparable; no mammalian treatment outcome	[102]
Device prevention	Acylase + amylase catheter coating	Coated urinary-catheter surface; static/dynamic biofilm and preventive animal catheter model	Selected assembly ≈30% greater antibiofilm performance than alternative coating; delayed colonization up to 7 days	Preventive surface experiment; not treatment of an established catheter infection	[3]
In vitro delivery	QSI + tobramycin nanoparticles	Squalenyl hydrogen sulfate formulation, PA14 biofilms/mucin, human cells and short zebrafish assessment	≈16-fold lower tobramycin requirement for tested in vitro biofilm eradication endpoint	Combination-delivery gain cannot be assigned exclusively to QS inhibition	[14]
In vitro delivery	Acylase + gentamicin nanospheres	Static/flow-associated biofilms, cell compatibility assays	Reported approximately 16-fold improvement in bactericidal activity versus bulk gentamicin; virulence-factor reduction 97 ± 1.8%	Antibiotic/carrier contribution, protein corona and absence of animal efficacy constrain attribution	[47]

Table 2 note. Enzyme concentration (mg/mL) describes a solution or in vitro assay condition, whereas administered dose describes the delivered mass (mg per animal), mass per body weight (ng/g or mg/kg), or both. For SsoPox-I, 250 µL × 1 mg/mL = 0.25 mg per rat in a single intratracheal instillation [21]; 0.5 mg/mL SsoPox-W263I refers only to in vitro exposure of clinical isolates [1,2]. Intranasal PvdQ ng/g values are body-weight-normalized administered amounts, not solution concentrations [20]. Animal infection, clinical-isolate laboratory, ex vivo, preventive device, and in vitro formulation studies are not equivalent levels of evidence. Numerical outcomes are study- and model-specific; missing experimental details are not inferred. Abbreviations: CF, cystic fibrosis; CFBE, cystic fibrosis bronchial epithelial; CFTR, cystic fibrosis transmembrane conductance regulator; CFUs, colony-forming units; PK, pharmacokinetics; QQ, quorum quenching; QS, quorum sensing; QSI, quorum-sensing inhibitor.

## 6. Evidence Limitations and Translational Challenges

### 6.1. Experimental Models and Evidence Assessment

Claims about quorum-quenching strategies should match the experimental model from which they are derived. Reporter systems such as *lasB-gfp*, *rhlA-gfp*, *pqsA-gfp* and related biosensors are useful for screening pathway activity, but reporter inhibition alone does not establish direct target engagement in native *P. aeruginosa* [118]. Chemical signal measurements, biochemical assays, binding or competition experiments, structural analyses, and genetic perturbations provide complementary levels of mechanistic evidence. QS-gene expression changes are contextual evidence, not proof of signal degradation or receptor inhibition. Table 3 lists a multidimensional set of endpoints for evaluating these claims.

Biofilm efficacy cannot be inferred from a single endpoint. Crystal-violet staining measures attached biomass, not viable-cell burden. MIC measures planktonic growth inhibition and does not substitute for minimum biofilm inhibitory concentration (MBIC), minimum biofilm eradication concentration (MBEC) or time-dependent killing measurements [17]. Mature-biofilm studies should specify biofilm age and evaluate viable-cell recovery, architecture, matrix properties, penetration, dispersion and eradication in parallel. Host experiments should separate bacterial burden, epithelial injury, cytokine responses, immune cell recruitment, tissue pathology, cell death and survival. Evaluating these endpoints separately helps prevent changes in reporter activity, biofilm biomass, or inflammation from being mistaken for bacterial eradication or clinical benefit.

### 6.2. Biological Variability and Therapeutic Escape

Anti-virulence interventions are not inherently resistance-proof. Experimental exposure to brominated furanone C-30 selected *P. aeruginosa* variants with reduced susceptibility linked to increased compound efflux; a mexR mutant was more pathogenic in a C. elegans assay in the presence of C-30 [119]. This directly observed example must be differentiated from pre-existing non-response in naturally QS-rewired or efflux-proficient isolates and from regulatory compensation that redirects virulence outputs after pathway perturbation.

Biological variability can produce primary non-response before treatment-driven resistance emerges. lasR-defective clinical isolates can retain or reconfigure downstream QS outputs and may adopt biofilm-adapted expression states [62,120], whereas environmental conditions can shift the functionally dominant branch [34]. PqsR inhibition can suppress selected Pqs-dependent phenotypes while leaving Rhl-associated outputs intact [93]. Baseline pathway dependence, strain background, and infection status may determine target relevance in a given isolate.

Selection pressure remains relevant to anti-virulence therapy [121]. MexAB-OprM-mediated efflux can markedly reduce the potency of QS inhibitors [12], whereas target mutation, altered signal production, pathway compensation and selection of strains that no longer depend on the targeted QS node are plausible escape routes. Social-evolution experiments suggest that community structure may constrain some forms of resistance [31]. Such experiments do not reproduce long-term exposure to clinically relevant compounds. Future resistance studies should use repeated treatments with pharmacologically relevant agents in chronic, spatially structured and clinically derived infection systems.

### 6.3. Pharmacological, Safety, and Translational Barriers

Chronic airway and wound infections may contain multi-species communities. The consequences of AHL or PQS interference for co-pathogens, cross-species chemical communication (including AI-2-associated processes), and microbiome recovery remain insufficiently established. Viability in multi-species communities, community composition, signal and metabolite profiles and longitudinal recolonization should be assessed alongside *P. aeruginosa* monoculture virulence endpoints [74,80,81,122].

Pharmacological translation requires evidence of active target-site exposure. Demonstrating lung deposition, intravesical localization, mucus penetration or nanoparticle accumulation does not establish an exposure–response relationship at the active target site [4,14,114,123]. Small-molecule candidates require characterization of permeability, efflux, solubility, local concentration, metabolism and target occupancy. Enzyme candidates require assessment of catalytic stability, substrate accessibility, tissue persistence, immunogenicity and activity in biological fluids. Repeated-dose tolerability, biodistribution, systemic leakage, sterilization stability and long-term toxicity remain insufficiently characterized.

Formulation benefits must be tested against physiological exposure barriers. Nanoparticle protein coronas can alter deposition and surface interactions; mucin binding, measured by adsorption and diffusion studies, may lower the free local concentration of hydrophobic QS inhibitors [14,15]. Repeated local administration of lactonases or other protein-based QQ agents requires measurement of catalytic stability in sputum and other biological fluids, persistence at the infection site, neutralizing antibody responses and local immunogenicity. Biofilm penetration measured in vitro cannot, by itself, resolve these uncertainties.

Microbiome and ecological effects are poorly characterized; none of the reviewed studies directly demonstrate microbiome preservation during QQ therapy. AHL degradation, receptor antagonism, PQS-associated dispersion and altered metabolic signaling can alter interactions among *P. aeruginosa*, commensal organisms, co-infecting species and host tissues, so ecological neutrality cannot be assumed [44,79,122]. Future studies should include polymicrobial communities, community composition and function, metabolite exchange, recovery after treatment and long-term selection for resistance or pathway escape.

### 6.4. Research Priorities and Translational Pathway

A proposed strain-informed selection workflow begins with genomic and functional characterization of patient-derived isolates. Sequence analysis of lasR/lasI, rhlR/rhlI, and pqsR/pqsA, together with assessment of pqsE and relevant efflux pathways, may identify candidate regulatory alterations but cannot independently establish pathway activity. Growth-normalized assays under infection-relevant conditions should measure 3-oxo-C12-HSL, C4-HSL, and HHQ/PQS alongside pathway-associated transcriptional and virulence outputs. Isolates displaying functional Las-dependent signaling may be evaluated using substrate-appropriate AHL-degrading enzymes or LasR antagonists. Persistence of RhlR-dependent outputs despite impaired Las activity supports further assessment of RhlR-directed interventions [120]. Where functional dependence is demonstrated, the PqsE–RhlR interface also merits investigation [27]. Evidence of PqsR-dependent transcriptional activity may support evaluation of PqsR antagonists, subject to confirmation of target engagement [10]. Candidate interventions should be examined in the corresponding clinical isolates for growth-independent activity, permeability and efflux limitations, and efficacy in established biofilms or host-mimetic models. When pathway dependence cannot be demonstrated, detection of a target gene alone should not justify target selection. This proposed workflow represents a testable framework for preclinical isolate stratification rather than a validated clinical diagnostic or treatment algorithm.

**Figure 4:**
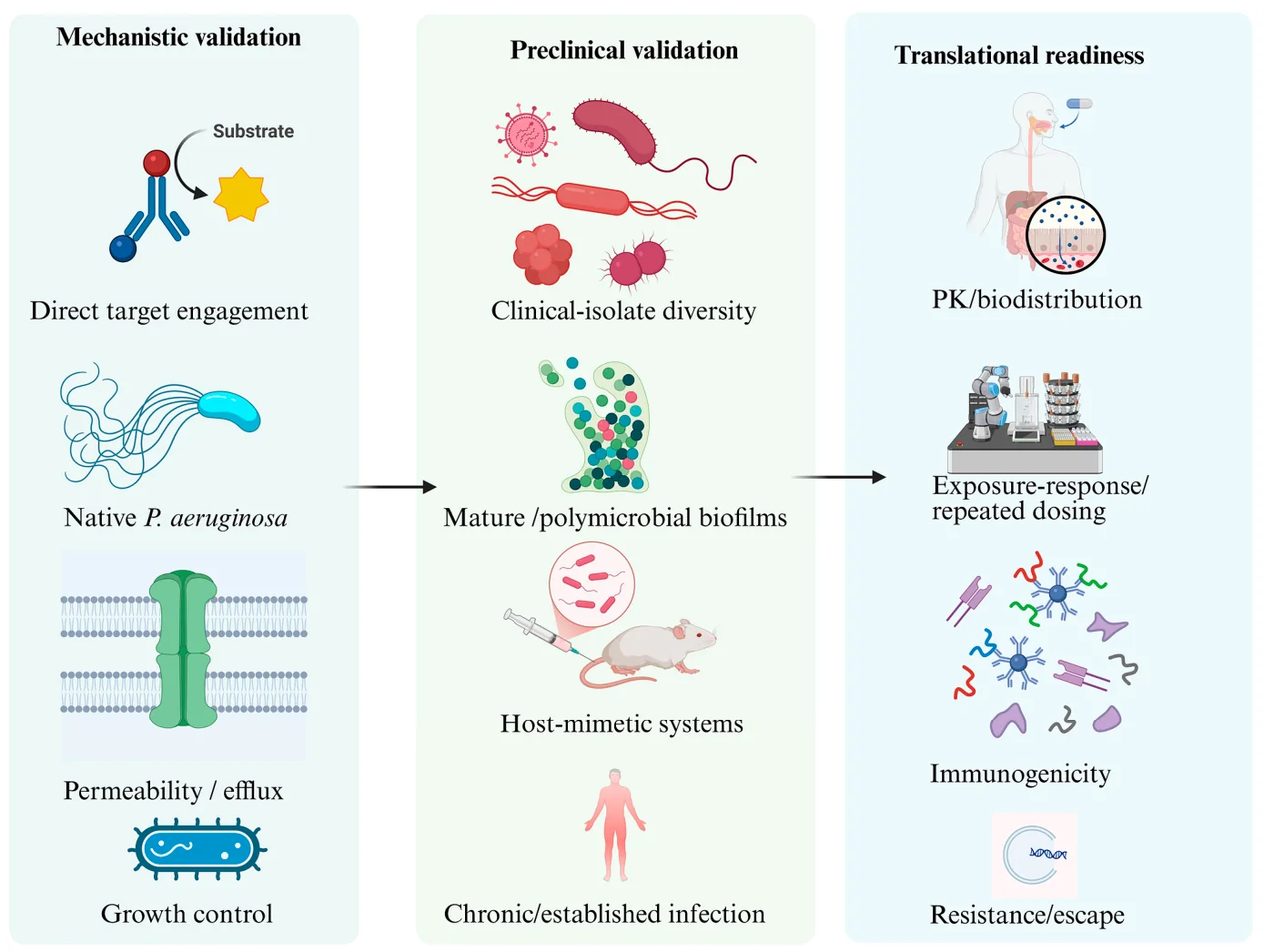
Translational-readiness requirements for quorum quenching and broader QS interference. The conceptual framework proceeds from mechanistic validation (direct target engagement, activity in native *P. aeruginosa*, permeability/efflux and growth controls), to preclinical validation (clinical-isolate diversity, mature and polymicrobial biofilms, host-mimetic systems and established infection), and then to translational assessment (pharmacokinetics/biodistribution, exposure–response and repeated dosing, immunogenicity, and resistance or pathway escape). Horizontal arrows represent research stages rather than a validated clinical decision algorithm. PK, pharmacokinetics.

Three areas warrant priority: molecular target validation, disease-relevant efficacy models, and pharmacology and safety. Molecular studies should prioritize targets that remain active in clinically relevant strains and assess direct target engagement alongside permeability, efflux, and growth controls. The PqsE–RhlR interface and rational multi-node strategies are relevant in settings where network bypass has been observed [57,96]. A structure-guided bifunctional aptamer that simultaneously blocks 3-oxo-C12-HSL and C4-HSL reduced rhamnolipid synthesis by 51.76%, pyocyanin production by 66.28% and biofilm formation by 74.46%, showing broad suppression across these measured QS-regulated outputs in the tested system [82]. Efficacy studies should move beyond early PAO1 assays to established, mature, polymicrobial and clinically derived biofilms and chronic mammalian infection models. Studies claim that therapeutic efficacy should begin treatment after infection is established. Pharmacology and safety studies should quantify target-site exposure, repeated dosing, immunogenicity, local toxicity, microbiome effects and long-term evolutionary responses. Table 4 summarizes evidence gaps and recommended study models; Figure 4 depicts requirements for translational readiness.

### 6.5. Polymicrobial Ecosystems and Host-Microbiome Considerations

*P. aeruginosa* frequently exists within polymicrobial communities in chronic airway and wound infections [124,125]. Accordingly, QS interference should not be evaluated solely in monoculture *P. aeruginosa* models. *P. aeruginosa* lacks canonical endogenous AI-2 production yet can sense AI-2 from neighboring species; PctA and TlpQ provide direct receptor-level evidence for this interspecies response [80,81]. Other interspecies signaling pathways may likewise affect interactions among co-colonizing microorganisms [126]. Although current QQ enzymes and receptor antagonists have primarily been characterized in *P. aeruginosa*-focused systems [48,49], their effects on non-target microbial communities and host-associated microbiota remain insufficiently defined. Future studies should incorporate polymicrobial models and microbiome-resolved analyses to determine whether QS interference provides selective pathogen suppression without disrupting beneficial microbial networks.

## 7. Conclusions

QQ and broader QS interference target distinct molecular processes in preclinical research on *Pseudomonas aeruginosa*. Reduced reporter activity, virulence output or biofilm biomass alone cannot identify the mechanism or predict host benefit. Among the interventions reviewed, AHL-degrading enzymes and selected PqsR inhibitors combine direct target evidence with host-level preclinical data, whereas the PqsE–RhlR interface is a biologically well-validated target but is not yet represented by a mature drug class. The strength and scope of evidence differ across intervention classes, as do substrate specificity, tissue access, isolate coverage and susceptibility to network bypass.

Efficacy varies across isolates, environments and treatment conditions. Network plasticity, LasR loss with retained Rhl activity, phosphate- or iron-dependent rewiring, efflux, delivery, formulation and mature-biofilm state can all alter response. Host outcomes and bacterial clearance often diverge, and antibiotic sensitization varies by agent, model and treatment timing. The most defensible near-term application is as an adjunct to standard treatment: candidate QQ or other QS-directed agents need direct target engagement, adequate delivery and testing in established infection models that measure viable burden, host injury, safety, pharmacokinetics, microbiome effects and evolutionary escape. Until those data are available, these approaches should be described as preclinical rather than clinically effective.

## Figures and Tables

**Table 3:** Evidence-informed criteria for multidimensional evaluation.

Evaluation Domain	Recommended Endpoint	Preferred Measurement	What It Demonstrates	Principal Confounder	Minimum Interpretation Requirement
QS target engagement	Direct signal or protein-target engagement	LC-MS/MS for AHLs/AQs; enzyme kinetics; ITC, crystallography, competition, pulldown, EMSA, mutant rescue	Claimed signal, enzyme, receptor, or interaction is affected	Reporter-only effects, docking, transcriptional change, non-native uptake	Pair direct measurement with native *P. aeruginosa*, dose response, counterscreens, growth controls
Bacterial growth	Growth and viability under exposure	OD600, CFU, MIC, time-kill, vehicle/inactive controls	Phenotype reduction is separable from antibacterial activity	Cytostasis, membrane damage, exposure mismatch	Measure growth/viability at the same concentration and time as QS endpoints
Biofilm biomass	Total attached material	Crystal violet or biomass staining plus parallel viability/matrix assays	Attached material changes	Dead cells or matrix can distort staining	Never infer eradication from biomass alone; pair with CFU and architecture
Viable burden	Living cells in culture, biofilm, tissue, or device	CFU per biomass/tissue/device/volume; detection limit; time-resolved recovery	Killing, clearance, or survival of viable cells	Aggregation, incomplete recovery, dispersion	Define recovery, detection limit, timing, and dispersed-cell handling
Matrix and architecture	Structure, matrix, penetration	Confocal/COMSTAT, EPS assays, QCM-D, SEM, penetration imaging	Remodeling or access within the biofilm	Biomass loss without killing; model-specific matrix	State biofilm age/model; pair imaging with burden and exposure
Dispersion	Release from biofilm	Flow-cell imaging, detached-cell CFU, PQS/OMV measures, post-dispersion killing	Developmental release or matrix escape	Dissemination risk or uncounted detached cells	Pair dispersion with killing, host outcomes, and post-dispersion burden
Virulence factors	Functional pathway output	Elastase, protease, pyocyanin, rhamnolipid, HAQ/PQS, HCN, secretion assays normalized to growth/CFU	Selected virulence attenuation	Reduced growth or cell-density effects	Normalize and link output to the claimed pathway
Antibiotic interaction	Additivity, synergy, antagonism, dose sparing	Checkerboard/response surface, time-kill, MBEC, mature-biofilm assays, monotherapy controls	Combination effect under defined timing	Direct antibiotic killing, carrier effects, early-biofilm bias	Include monotherapies, inactive/target-deficient controls, mature biofilms, predefined synergy rule
Epithelial barrier	Barrier function and epithelial injury	TEER/permeability, CFTR localization/function, tight-junction markers, repair, viability	Host barrier protection or restoration	Cell-line specificity, secretome effects, direct host drug activity	Pair barrier readouts with burden, host toxicity, bacteria-free controls
Inflammation	Cytokines and leukocyte recruitment	BAL/tissue cytokines, neutrophils, macrophages, MPO, histology	Immune/inflammatory modulation	Immunosuppression can mimic protection	Distinguish reduced injury from impaired defense using burden, pathology, survival
Oxidative stress	Host redox injury/protection	ROS, lipid/protein oxidation, antioxidant state, tissue iron, signal concentration	Direct host oxidative injury or protection	Bacterial iron/QS changes are not host ROS	Measure host oxidative damage directly and link to tissue signal exposure
Cell death	Apoptosis, necrosis, membrane injury	Caspases, TUNEL, annexin V, LDH, orthogonal viability assays	Host-cell death pathway change	Assay interference, metabolic suppression	Use orthogonal assays and separate direct host from secretome effects
Pathology	Tissue injury or repair	Blinded histology, imaging, lesion size, burden/inflammation correlation	Tissue-level protection	Observer bias, burden-driven pathology	Use blinded scoring and correlate with burden, inflammation, exposure
Survival/relapse	Composite host benefit	Kaplan–Meier survival, humane endpoints, relapse/recolonization, timing metadata	Survival benefit under tested timing	Prophylactic or early dosing overstates treatment relevance	Report timing, established-infection status, burden, relapse, pathology
PK/local exposure	Active target-site exposure	LC-MS or validated activity assay in plasma, BAL, mucus, wound, urine, tissue; exposure-response	Agent reaches target at active concentration	Fluorescence or deposition does not establish active exposure	Measure active concentration over time and link to target engagement/outcome
Toxicity/immunogenicity	Local/systemic tolerability and immune response	Cell viability, organ histology, serum markers, complement/cytokines, anti-drug antibodies, repeated dosing	Safety under relevant exposure	Short exposure, cell-line-only assays, formulation effects	Include vehicle, inactive agent, repeated-dose, organ, and immune controls
Microbiome/ecology	Community selectivity and recovery	Longitudinal amplicon/metagenomics, metabolomics, commensal growth, recovery sampling	Ecological preservation or disruption	Single-species models and no baseline/recovery	Use host-associated communities with pre-, during-, post-treatment sampling
Resistance/escape	Target loss, efflux, compensation, durable resistance	Serial passage, repeated treatment, MIC/phenotype tracking, WGS, target/signal sequencing, competition	Evolutionary durability or bypass	Idealized mimics may not reproduce clinical evolution	Use clinically relevant agents, native isolates, sequencing, fitness, social-ecology endpoints

**Table 4:** Translational barriers and research priorities.

Evidence Gap	Why It Matters	Current Evidence	Recommended Model or Method	Recommended Endpoint
Chronic infection models	Chronic infection selects rewiring, mucoidy, persistence, and altered response	Acute or treatment-at-challenge studies dominate; M64 persistence evidence exists	Chronic/recurrent pulmonary, wound, urinary, device models using clinical/mucoid isolates after establishment	Target engagement, viable burden, relapse, pathology, inflammation, survival, exposure response
Mature/polymicrobial biofilms	Mature matrices and neighboring species alter penetration, dispersion, tolerance, antibiotic response	Mature single-species and host-mimetic models exist; polymicrobial and chronic models remain scarce	Mature flow biofilms, artificial sputum/wound fluid, polymicrobial communities, host-mimetic matrices	Biomass, viable burden, matrix, penetration, dispersion, eradication, antibiotic interaction
Clinical isolates/QS rewiring	LasR loss, retained Rhl activity, mucoidy, isolate-specific responses determine target coverage	Clinical collections and isolate panels informative; mammalian intervention testing limited	Stratified *lasR*-defective, Rhl-active, mucoid, MDR, CF, wound, urinary, device isolate panels	Signal profile, target dependence, virulence response, biofilm phenotype, responder class
Direct synthase and PqsE–RhlR inhibitors	Genetic vulnerability does not establish druggability or exposure	PQS-synthesis/RhlI evidence exists; PqsE–RhlR biology well supported; no established interface inhibitor	Purified protein plus native permeability, efflux, target-mutant, rescue studies	Binding/kinetics, exposure, occupancy, pathway output, growth separation
Reproducible antibiotic interaction	Adjunctive positioning requires reliable benefit and documented failures	Positive and negative results coexist; no universal sensitization rule	Pre-registered combination matrices, time-kill, mature-biofilm MBEC, target-deficient controls	Synergy, antagonism, dose sparing, viable burden, relapse, isolate reproducibility
PK and biodistribution	Reporter activity does not prove active tissue exposure	Deposition, mucus penetration, intravesical localization explored; full profiles absent	LC-MS/activity assays and quantitative imaging in plasma, BAL, mucus, wound, urine, tissue	Concentration-time, half-life, active fraction, target-site/off-target distribution, exposure response
Repeated dosing and immunogenicity	Enzymes, small molecules, and carriers may lose activity or cause cumulative toxicity	Short-course, prophylactic, acute studies dominate; systematic immunogenicity absent	Repeated-dose mammalian efficacy/toxicology; enzyme/carrier immunology	Accumulation, activity loss, organ effects, anti-drug antibodies, complement, cytokines, efficacy loss
Microbiome safety	QS interference may alter commensals, co-infection, metabolites, spatial ecology	Direct microbiome preservation or toxicity evidence absent	Longitudinal polymicrobial airway, wound, urinary, device communities with recovery phase	Community composition, commensal function, metabolome, co-infection behavior, recovery
Resistance/evolutionary escape	Target loss, efflux, compensation, social selection could reduce durability	Efflux, natural rewiring, idealized social-evolution barriers documented	Serial passage, repeated-treatment evolution, WGS, target/signal sequencing, efflux assays, competition	MIC/tolerance shift, target loss, bypass, efflux, fitness cost, social spread, re-sensitization
Biomarkers/patient selection	Pathway-specific adjuncts require active target identification	BALF/isolate QS phenotyping suggestive; no validated pharmacodynamic biomarker	Prospective AHL/AQ, receptor, PqsE/RhlR, mucoidy, biofilm-state measurement; adaptive cohorts	Pathway suppression linked to response; responder rate by QS state, site, stage, history
Clinical validation	Preclinical host protection and delivery do not establish patient benefit	No retained study demonstrates patient-treatment efficacy or routine clinical use	Manufacturing, toxicology, PK, controlled adjunctive trials in biomarker-selected populations	Safety, pharmacodynamics, bacterial burden, relapse/exacerbation, antibiotic exposure, patient outcomes

Abbreviations: AHL, N-acyl homoserine lactone; AQ, alkylquinolone; BAL, bronchoalveolar lavage; BALF, bronchoalveolar lavage fluid; CF, cystic fibrosis; LC-MS, liquid chromatography–mass spectrometry; MBEC, minimum biofilm eradication concentration; MDR, multi-drug-resistant; PK, pharmacokinetics; QS, quorum sensing; WGS, whole-genome sequencing.

## Data Availability

No new data were created or analyzed in this study. Data sharing is not applicable to this article.
